# Supplementary material for: Ultrafast electronic response of graphene to a strong and localized electric field
Source: Nat Commun. 2016 Dec 21;7:13948. doi: 10.1038/ncomms13948 (PMC5187589; doi:10.1038/ncomms13948)
Supplement: Supplementary Information — Supplementary Figures, Supplementary Notes and Supplementary References. [file ncomms13948-s1.pdf]

Supplementary note 1: Target preparation and Characterization

A challenging part of the experiment is the preparation of a macroscopic, freestanding single layer of high quality graphene. The standard approach is to transfer graphene, grown on a copper foil via chemical vapour deposition (CVD), onto a grid used for transmission electron microscopy (TEM) with the help of a thin layer of polymethylmethacrylat (PMMA) for mechanical stabilization[1]. Later, the PMMA layer has to be removed and this always constitutes a source of severe contamination[2]. The use of PMMA is therefore avoided entirely, and instead the sample preparation procedure from[3] is adapted.

A 300-mesh gold TEM-Grid with a quantifoil (regular array of 1.2 μm holes) on top was used as the graphene carrier. Cross-check using highly charged ion transmission experiments with bare (i.e. without graphene coverage) 300-mesh gold quantifoil TEM grids confirmed that ions were fully neutralized in the Cu/Au grid bars as well as the tens of nm thick quantifoil, or were transmitted unhindered through the hole arrays. Thus any measured signal from the TEM grids with graphene spanning over the grid holes exclusively probes the intrinsic nature of the suspended graphene membrane. The grid was placed onto commercially available CVD graphene (Graphenea). Isopropyl alcohol was used to increase the adhesion between the quantifoil and the graphene layer. To remove the copper foil, the stack was placed onto the surface of an ammonium persulphate solution. After approximately 24 hours the etchant was substituted by deionized water and the TEM-Grid was carefully lifted out of the fluid. To remove the contaminations introduced during this procedure, we embedded the transferred graphene on TEM grid into active carbon within a ceramic dish and then heated it in an oven. The temperature of the samples was increased with a heating rate of $\approx 300°\mathrm{C\,h^{-1}}$ to a maximum temperature of 260°C. This temperature was held for 30 min, and then the sample was let to cool down. Reaching $\approx 70°$C, we took the sample out of the active carbon. Finally, a smooth nitrogen stream was used to remove the remaining active carbon from the grid.

The quality of the transferred graphene was checked by Raman spectroscopy. A typical Raman spectrum is shown in Supplementary Figure. 1a. The FWHM of the 2D mode peak provides the number of layers of the graphene sample. The inspected graphene samples show a FWHM of the 2D peak between 26-40 $\mathrm{cm^{-1}}$ and thus indicate single layer graphene (bilayer graphene would have a FWHM of $>50\,\mathrm{cm^{-1}}$). From the ratio of the integrated intensities of the D and G mode peaks the amount of defects can be evaluated and is negligibly small for the prepared graphene sheets. On the basis of these Raman data, we additionally determined that typically 80-90% of the Quantifoil holes are covered with graphene.

To make a rough estimate of the contamination of the graphene sheets, scanning transmission electron microscopy (STEM) measurements have been performed (see Supplementary Figure 1b) using a Nion UltraSTEM100 at 60 kV electron acceleration voltage and using high-angle annular dark field (HAADF) imaging. They show clean monolayer graphene regions of up to hundreds of nm dimensions in lateral extension, surrounded by graphene regions covered with a meandering network of thin hydrocarbon adsorbates (equivalent in thickness to up to $\sim$6 graphene layers, as estimated by HAADF intensities in STEM) and very few minor and isolated metal residues (presumably from the CVD and transfer processes). We note that despite the careful PMMA-free sample preparation and the activated carbon dry cleaning, such a low level of residual hydrocarbon adsorbates can result from air exposure of samples between fabrication and measurements and is typical for the state-of-the-art of suspended graphene membrane[3–5]. Effects of electron beam exposure on the contamination morphology was minimized in our STEM measurements via acquisition of fast snapshot images and use of a very low base pressure in the STEM system ($\sim 10^{-9}$ mbar).

Supplementary note 2: TDDFT calculations

Generalities.

To study the electron dynamics in collisions of the highly charged ions (HCI) with graphene we use the Kohn-Sham (KS) scheme within Time Dependent Density Functional Theory (TDDFT)[6–8]. The use of

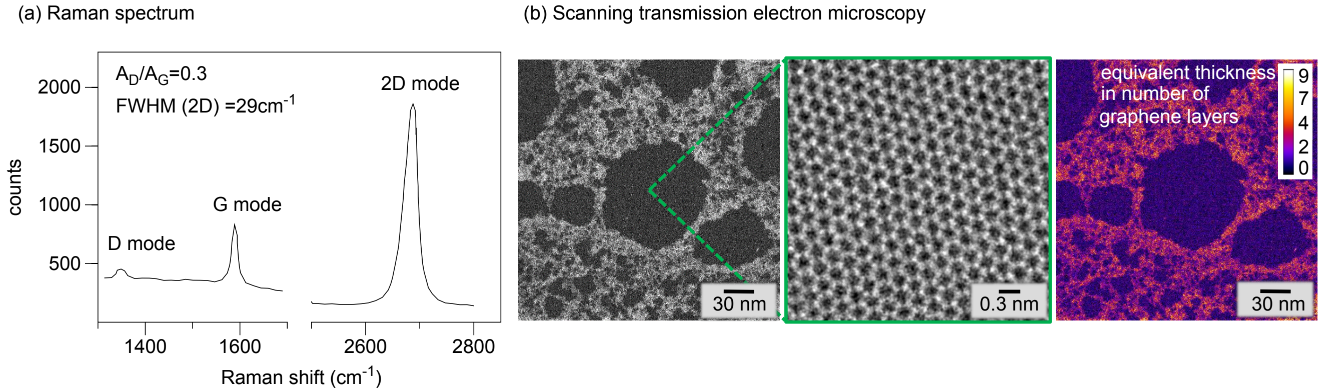

Supplementary Figure 1. Raman spectrum and STEM measurement
a) Raman spectrum of a graphene sample; the FWHM of $\sim 30\,\mathrm{cm}^{-1}$ of the 2D mode peak ensures single layer graphene and the ratio of the integrated intensities of the D and G mode peaks points to a negligible amount of defects b) STEM measurements (left) show clean monolayer graphene regions with a lateral extension of up to hundreds of nm, surrounded by graphene regions covered with thin hydrocarbon adsorbates. The monolayer nature of the clean graphene regions was confirmed using atomic-resolution STEM imaging (middle). Using the roughly linear intensity scaling of the HAADF STEM signal with specimen thickness, and assuming as an upper limit the same density for the hydrocarbon adsorbates as for graphene, a thickness estimated for the hydrocarbon contamination was calculated as equivalent thickness in number of graphene layers (right). This analysis shows that the hydrocarbon deposits on the graphene have a thickness corresponding to $\sim$3 graphene monolayers with some regions on the suspended graphene basal planes reaching up to $\sim$6 layers.

TDDFT allows to consistently address the one-electron and the multi-electron processes, as well as to follow the real time evolution of the electron densities, currents, and fields in the system. In a number of recent publications, for the case of the negative, neutral, single and double charged projectiles, TDDFT has been shown to provide an ab initio description of the stopping power of ions in solids[9–12], as well as the energy loss in transmission through graphene[13–17].

The specific details on the present implementation of the TDDFT can be found elsewhere[9, 18]. In brief, the time-dependent electron density of the many-body system $n(\mathbf{r},t)$ is represented via the density of a fictitious system of non-interacting electrons, the Kohn–Sham system, given by:

$$n(\mathbf{r},t) \;=\; \sum_{j\in occ} \left|\psi_j(\mathbf{r},t)\right|^2 \qquad \text{(Supplementary Equation 1)}$$

with the summation running over all the occupied KS orbitals $\psi_j(\mathbf{r},t)$. The time evolution of the KS orbitals is governed by the time-dependent Scrödinger equation:

$$i\frac{\partial\psi_j(\mathbf{r},t)}{\partial t} \;=\; \{T+V_{\text{eff}}[n(\mathbf{r},t)]\}\,\psi_j(\mathbf{r},t). \qquad \text{(Supplementary Equation 2)}$$

Here, $T=-\frac{1}{2}\Delta$ is the kinetic energy operator, and $V_{\text{eff}}[n(\mathbf{r},t)]$ is the effective KS potential that depends on the electron density.

$$V_{\text{eff}}[n(\mathbf{r},t)] = V_{\text{H}}[n](\mathbf{r},t)+V_{\text{xc}}[n(\mathbf{r},t)]+V_{\text{ion}}(\mathbf{r},t). \qquad \text{(Supplementary Equation 3)}$$

The Hartree $V_{\rm H}[n(\mathbf{r},t)]$ and exchange-correlation $V_{\rm xc}[n(\mathbf{r},t)]$ potentials are generally time-dependent through the time-dependence of the electron density. The exchange-correlation potential is described within the adiabatic local density approximation (ALDA)[6,8,19] using the exchange-correlation kernel of O. Gunnarsson and B. I. Lundqvist[20].

At this point it is worth to stress that the ALDA with exchange-correlation kernels developed so far in quantum chemistry does not allow to describe the Auger processes involving strong binary electron-electron interactions with large energy exchange. Thus, strictly speaking, the present approach is well suited for the incoming part of the multicharged ion trajectory, where a strongly excited hollow atom is formed via multielectron capture from graphene towards loosely bound orbitals of the projectile. The relaxation of the hollow atom via Auger cascades towards inner electronic shells is beyond the reach of the present description. We further address this point below in this Supplementary note.

The last term in Supplementary Equation 3 describes the electron interaction with the projectile ion core. We consider a fully stripped ion of charge $q_{\rm in}$ impinging at graphene along the straight line trajectory $\mathbf{R}(t) = \mathbf{R_0} - \mathbf{v}t$ perpendicular to the graphene layer. $\mathbf{R}(t)$ defines position ion core. Since the calculated energy loss is much smaller than the kinetic energy, the projectile speed $|\mathbf{v}|$ is set constant during the collision. With these assumptions $V_{\rm ion}(\mathbf{r},t) = V_{\rm ion}(|\mathbf{r}-\mathbf{R}(t)|)$. To avoid the divergence of $V_{\rm ion}$ at $\mathbf{r} = \mathbf{R}(t)$, the positive charge $q_{\rm in}$ of the ion core is homogeneously distributed over the small sphere of radius $R_\rho(q_{\rm in})$ given by $(R_\rho(q_{\rm in})/\rho)^3 = q_{\rm in}$. This is equivalent to the description of the projectile as a small highly charged quantum dot or, equivalently, a fully ionised jellium metal (JM) cluster[21,22] characterised by the density parameter ρ. We have used $\rho = 1$ a$_0$ and $\rho = 0.5$ a$_0$ (a$_0$ = 0.53 Å is a Bohr radius) so that (in units of Bohr radius):

$$\begin{aligned} R_1(q_{\rm in}) &= \sqrt[3]{q_{\rm in}}, \\ R_{0.5}(q_{\rm in}) &= \sqrt[3]{q_{\rm in}}/2. \end{aligned} \qquad \text{(Supplementary Equation 4)}$$

For example, when $q_{\rm in} = 20$ the corresponding radii are $R_1(20) = 1.43$ Å and $R_{0.5}(20) = 0.72$ Å, respectively so that the projectile considered here is very compact. Within the Jellium cluster model, the $V_{\rm ion}$ potential is given by:

$$\begin{aligned} V_{\rm ion}(\zeta) &= -\frac{q_{\rm in}}{\zeta}, \qquad \zeta \geq R_\rho(q_{\rm in}), \\ &= -\frac{q_{\rm in}}{2\zeta}\left[3 - \left(\frac{R_\rho(q_{\rm in})}{\zeta}\right)^2\right], \qquad \zeta < R_\rho(q_{\rm in}), \end{aligned} \qquad \text{(Supplementary Equation 5)}$$

where $\zeta = |\mathbf{r} - \mathbf{R}(t)|$. It follows from Supplementary Equation 5 that $R_{1/0.5}(q_{\rm in})$ corresponds to the cutoff parameter for the Coulomb potential of the HCI.

This simple picture of a HCI suffices to model the electron capture onto the outer Rydberg states of the projectile with hollow atom formation, as well as the energy loss and the electronic response of graphene, which is the purpose of this paper. The inner shell structure of the Xe projectiles is not described with this model consistent with impossibility to describe electronic relaxation to these shells with available exchange-correlation potentials.

In the TDDFT simulation of the HCI interaction with graphene one might use the supercell geometry or finite size of a graphene patch as has been done in the ab initio studies of the energy loss of the singly and doubly charged projectiles[14–16]. However, the peculiarity of the projectile-surface charge transfer involving the HCI is that because of the large attractive potential, the projectile starts to capture the electrons far away from the graphene sheet. The strongly polarized hollow atom is formed with electrons occupying diffuse Rydberg orbitals. This requires that the computational mesh is large enough to cover an

extended region of space in the direction perpendicular to the surface, and in the lateral direction. The former is needed to correctly capture the onset of the electron transfer and the latter guaranties that the periodic boundary conditions or the finite size effects do not alter the results of the calculations. Along with this difficulty, considering the large number of electrons transferred to the projectile and emitted into vacuum by ionization because of the HCI impact, the fraction of the graphene surface per projectile has to be sufficiently large to avoid charging effects which would block the electron transfer. All together these constraints render the calculations with a full atomistic representation of graphene computationally extremely demanding, if feasible at all. We, therefore, decided to adopt the JM model to describe the graphene valence electrons.

The JM description has been successfully used previously to describe plasmon resonances, photoemission, photoabsorption and strong field effects in C_{60} clusters[23–28]. Following these approaches we model graphene as a conductive layer of width $\delta = 1.484\,\text{Å}$. The carbon ion cores are represented by a uniform positive background charge with the density $n_{Gr} = \left[\frac{4\pi}{3} r_s^3\right]^{-1}$. The density parameter is set as $r_s = 0.615\,\text{Å}$ so that in the neutral system we account for the 4 valence electrons per carbon atom in the unit cell. We consider finite size jellium model disks of diameter D comprising 500 ($D = 20.458\,\text{Å}$), 1004 ($D = 28.938\,\text{Å}$), and 2000 ($D = 40.81\,\text{Å}$) electrons which allows to check the convergence of the results with respect to the graphene patch size. Cylindrical coordinates (ρ, ϕ, z) are used in the TDDFT calculations with the projectile moving along the z-axis, which is set perpendicular to the surface of the disk and containing the disk center. In cylindrical coordinates, the density of the positive background charge is given by

$$n_+(\mathbf{r}) = \left[\frac{4\pi}{3} r_s^3\right]^{-1} \Theta(\delta/2 - |z|)\Theta(D/2 - \rho), \qquad \text{(Supplementary Equation 6)}$$

where Θ is the Heaviside step function. In order to impose the correct work function of graphene $\Phi = 4.6$ eV,[29,30] a constant attractive potential $V_{stab} = 13.6$ eV is added to the $V_{\mathrm{H}}[n(\mathbf{r},t)]$ potential inside the jellium disk. This is the so-called "stabilized jellium model"[31].

With the KS orbitals represented on a mesh in cylindrical coordinates, Supplementary Equation 2 are solved using real-time propagation similar to that developed for the one-electron wave packet propagation in cylindrical coordinates and detailed elsewhere[32–34]. To avoid complications linked with the change of the number of electrons in the computation box we do not introduce the complex absorbing potentials (CAPS)[35] at the boundaries of the mesh. Since the mesh used in our calculations is large, the overall density change due to the emitted electrons distributed over the computational box remains very small.

Obviously, within the JM the motion of graphene lattice atoms is not considered. We thus calculate the electronic contribution to the projectile energy loss, which has been shown to be a dominant energy loss channel for the projectile speeds considered here[13]. The energy loss of the projectile ΔE_P is calculated integrating the action of the Hellmann-Feynman force F_z over the trajectory

$$\Delta E_P = \int F_z \, v \, dt. \qquad \text{(Supplementary Equation 7)}$$

The Hellmann-Feynman force is obtained from the time dependent electron density as

$$F_z = -\frac{d}{dZ} \int d^3\mathbf{r}\, [n(\mathbf{r},t) - n_+(\mathbf{r})]\; V_{\text{ion}}(\mathbf{r} - \mathbf{R}), \qquad \text{(Supplementary Equation 8)}$$

where Z is the projectile z-coordinate. Observe that, because of the symmetry of the problem, the only non zero component of the force is along z-axis. Our scheme preserves the total energy of the quantum-classical

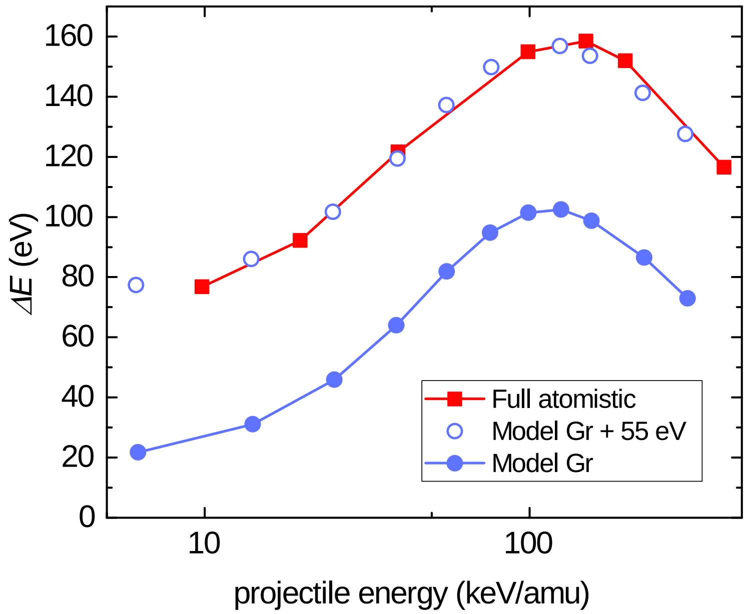

Supplementary Figure 2. Energy loss of a He^{++} projectile traversing graphene
Electronic contribution to energy loss of the He^{++} projectiles traversing graphene at normal incidence. The data are presented as function of the projectile energy expressed in keV per atomic mass unit. Red line with squares: full atomistic TDDFT calculation in the supercell geometry from[15]; Blue line with circles: present TDDFT study with JM model of graphene; Open blue circles: Present TDDFT results shifted by +55 eV.

system[36] $\mathscr{H} = Mv^2/2 + E_{el}$. Here M is the projectile mass, and the electronic (quantum) energy part E_{el} is given by

$$\begin{aligned} E_{el} &= \sum_{j \in occ} \langle \psi_j(\mathbf{r},t)|T|\psi_j(\mathbf{r},t)\rangle \\ &+ \int d^3\mathbf{r}\,[n_+(\mathbf{r}) - n(\mathbf{r},t)]\,\{[V_{\rm ion}(\mathbf{r},t) + V_{stab}(\mathbf{r}) + 0.5\;V_{\rm H}[n(\mathbf{r},t)]\} \\ &+ \int d^3\mathbf{r}\; n(\mathbf{r},t)\;\varepsilon_{XC}[n](\mathbf{r}), \end{aligned} \quad \text{(Supplementary Equation 9)}$$

where ε_{XC} is the exchange-correlation energy density[20,37]. As a consequence, ΔE_P can be alternatively obtained as $\Delta E_P = -[E_{el}(t=\infty) - E_{el}(t=0)]$.

At the beginning of the collision the projectile is assumed to be fully ionized, and the initial conditions for the time dependent Supplementary Equation 2, $\psi_j^0(\mathbf{r}) \equiv \psi_j(\mathbf{r}, t=0)$, correspond to the KS orbitals of the ground-state graphene disk. Therefore, prior to the TDDFT calculations, the standard density functional theory (DFT) study has been performed. The KS orbitals $\psi_j^0(\mathbf{r})$ satisfy the stationary Kohn Sham equations:

$$\begin{aligned} \{T + V^0_{\rm eff}[n^0(\mathbf{r})]\}\;\psi_j^0(\mathbf{r}) &= E_j\psi_j^0(\mathbf{r}), \\ n^0(\mathbf{r}) &= \sum_{j\in occ} \left|\psi_j^0(\mathbf{r})\right|^2 \end{aligned} \quad \text{(Supplementary Equation 10)}$$

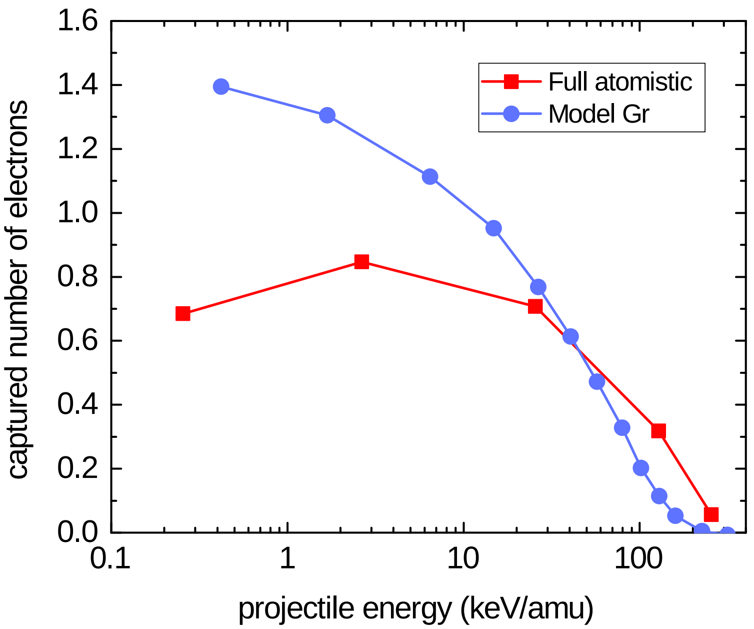

Supplementary Figure 3. Captured electrons by a He^{++} projectile traversing graphene
Mean number of electrons captured by the He^{++} projectiles after collision with graphene at normal incidence. The data are presented as function of the projectile energy expressed in keV per atomic mass unit. Red line with squares: full atomistic TDDFT calculation in the supercell geometry from[16]; Blue line with circles: present TDDFT study with JM model of graphene.

where E_j is the energy of the K-S orbital. The effective potential

$$V^0_{\mathrm{eff}}[n^0(\mathbf{r})] = V^0_{\mathrm{H}}[n^0(\mathbf{r})] + V^0_{\mathrm{xc}}[n^0(\mathbf{r})] \qquad \text{(Supplementary Equation 11)}$$

is time independent. We use superscript ^{0} to refer to the ground state of the system.

Within the JM the electronic structure of the free-standing graphene layer is characterised by the two continua of states. The electron motion is quantized in z-direction perpendicular to the layer. The electron motion parallel to the layer is free, and it is characterized by the wave vector $\mathbf{k}_{||}$. The energies of the electronic states are given by:

$$E_{1/2}(k) = E_{1/2} + k^2_{||}/2, \qquad \text{(Supplementary Equation 12)}$$

where $E_1 = -31.95$ eV and $E_2 = -13.68$ eV. These bands mimic the σ- and π-band of graphene, respectively. The electronic states $\psi^0_j(\mathbf{r})$ of the model graphene disk originate from the quantization of these two free electron continua by the finite lateral size.

A remark is in order here concerning the JM used for graphene. Taking into account an extremely strong Coulomb potential of the ingoing projectile, the electronic structure of graphene is locally modified. Thus, even though the JM does not reproduce the details of the stationary band structure of pristine graphene including the Dirac cone at K-point, we believe that it allows to capture the essential physics of the dynamics of the HCI-surface charge transfer and energy loss.

To test the effect of the JM approximation on the actually measured quantities we performed calculation of the energy loss of He^{++} projectiles and compared it with available TDDFT results obtained recently

with full atomistic description of the graphene layer[15,16,38] as we show in Supplementary Figure 2. In overall, the projectile energy dependence of the electronic stopping obtained in full atomistic TDDFT study is well reproduced within our model approach. In particular, we find the correct energy position of the energy loss maximum as well as the overall shape of the energy dependence. However, as follows from the results presented with open circles in Supplementary Figure 2, the JM systematically underestimates the energy loss value by ≈ 55 eV. We attribute the lower energy loss calculated with the present model to the higher values of the binding energies $|E_1| = 31.95$ eV and $|E_2| = 13.68$ eV states as compared to the σ ($|E_\sigma| = 20$ eV) and π ($|E_\pi| = 7$ eV) bands of graphene[16]. In particular, at low projectile velocities when weakly bound π electrons dominate the electronic stopping, this effect is more significant.

These arguments are further supported by the analysis of the final charge state of the He-projectile after the collision with graphene layer at normal incidence shown in Supplementary Figure 3. The mean number of electrons captured by the He^{++} incident ion is determined on the exit part of the trajectory after the projectile has traversed the graphene layer. It is obtained integrating the electron density within the sphere of 2.65 Å around the projectile. Even though this procedure is focused on the ground state population analysis and does not allow to account for the occupation of the diffuse excited orbitals, we apply it here for consistency with[16]. While at high projectile energies the JM and full atomistic TDDFT calculations agree quantitatively, qualitative differences emerge below 20 keV/amu. A decrease in the probability of electron capture with lowering energy is obtained in full atomistic calculations, while the present JM shows larger neutralisation rates at lower energies. This result is consistent with the higher binding energies of the electronic states of graphene in the present model. For example, the binding energy of the $E_1(k)$ band is resonant with the first ionization potential of He allowing the resonant electron capture by He^+ projectile, while the σ and π bands of graphene have lower binding energies and electron capture into inner projectile levels is then a nonadiabatic process requiring high enough projectile speed.

TDDFT Results for the highly charged ions.

The number of electrons captured by the HCIs $N_{\rm cap}$ is shown in the left panel of Supplementary Figure 4 for the $q_{\rm in}$=10, 20 and 40 projectiles incident at the graphene layer represented with 1004 electrons jellium disk. $N_{\rm cap}$ is determined on the outgoing trajectory path after the projectile traversed the target. It is obtained by integrating the electron density within the sphere centered at the projectile. The radius of the sphere is set as 12.5 Å, i.e. it is sufficiently large to account for the occupation of excited states. Within the present approach, $N_{\rm cap}$ appears to be quite independent of the projectile velocity. Consistent with experimental observations we obtain that the HCI with initial charge $q_{\rm in}$ is almost neutralized in all cases with residual charge as small as $q_{\rm in}/10$. In the right panel of Supplementary Figure 4 we show the total number of electrons lost by the target $N_{\rm lost}$ (ionized electrons). It is approximately twice larger than $N_{\rm cap}$ so that $N_{\rm vac} = N_{\rm lost} - N_{\rm cap} \simeq N_{\rm cap}$ electrons are emitted into the free-electron continuum upon impact. The slower is the projectile, the more adiabatic is the interaction. The electron emission $N_{\rm vac}$ then decreases leading to the overall decrease of the number of ionized electrons for ion velocities lower than 0.44 nm/fs.

In the experiments, slower projectiles capture more electrons leading to the velocity dependent $N_{\rm cap}$. Indeed, the slow collision allows full relaxation of the projectile into the ground state via Auger processes. As discussed above, the present theoretical approach does not allow to include Auger processes. It is aimed at the description of the transfer of valence electrons between the HCI and graphene.

In Supplementary Figure 5 we analyse the dependence of the calculated energy losses on the model used to describe the HCI projectile, and on the initial charge of the projectile $q_{\rm in}$. As follows from the results shown in Supplementary Figure 5, reducing the cutoff parameters for Coulomb potential from $R_1(q_{\rm in})$ to $R_{0.5}(q_{\rm in})$ i.e. representing the projectile core as more compact, leads to the overall increase of the calculated energy loss. For the present case we calculated that a reduction of cutoff radius by a

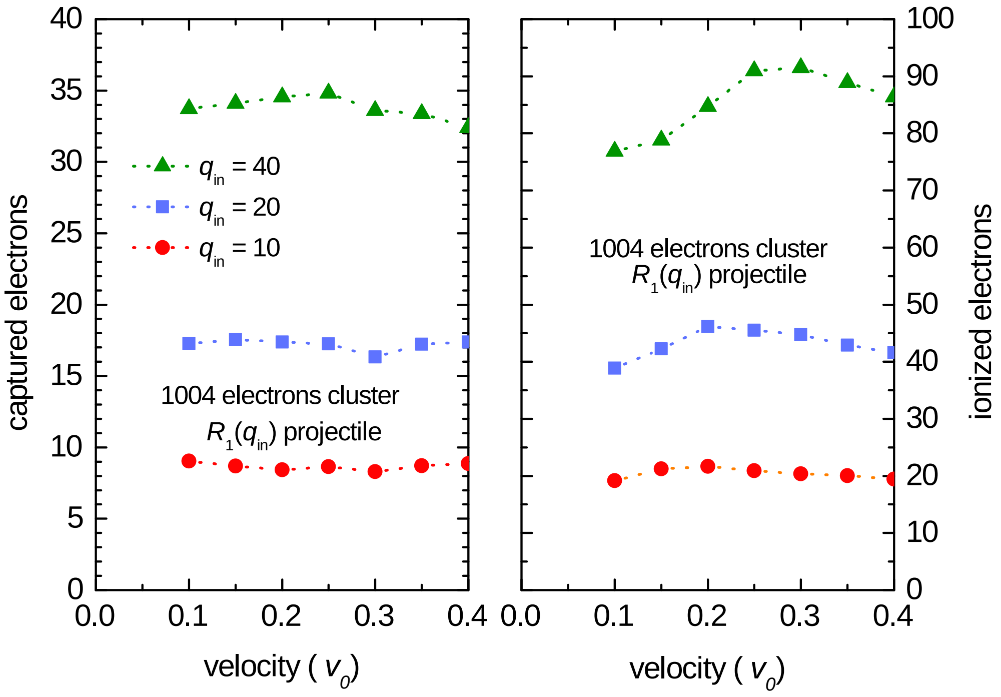

Supplementary Figure 4. Captured electrons by the HCI and ionized electrons from graphene Number of captured electrons by the HCI (left panel) and ionized electrons from the graphene layer (right panel) for incident charge states q_{in}=10, 20 and 40 as a function of the incident velocity. The projectile has been modeled using $R_1(q_{\text{in}})$ cutoff radius for Coulomb potential as defined by Supplementary Equation 4. Results are shown for the graphene layer represented with 1004 electrons jellium disk. Lines are guides to the eye.

factor of two leads to the twice larger energy losses. This increase can be even more prominent at higher velocities and charge states. We argue that, together with higher binding energies of the graphene bands within the present jellium model, the sensitivity to the Coulomb potential cutoff is at the origin of the underestimation of the measured energy losses by present calculations as shown in Figure 3 of the paper.

The dependence of the projectile energy loss ΔE_P on the incident charge state q_{in} is shown in right panel of Supplementary Figure 5. For the fixed model of the projectile, the quadratic dependence of $\Delta E_P \sim q_{\text{in}}^2$ is found in full accord with experimental data.

The target size dependence of the calculated energy loss ΔE_P and the number of captured electrons N_{cap} is analysed in Supplementary Figure 6. The lateral size of the jellium disk D was varied by a factor of two (variation of the number of electrons by a factor of four from 500 to 2000). The results presented in Supplementary Figure 6 show that the calculations converged with respect to the finite size effects already for a 500 electrons jellium disk as far as the projectiles with $q_{\text{in}} = 10$ and 20 incident charge states are concerned. For the highest incident charge state considered here, $q_{\text{in}} = 40$, finite size effects are still present, although they do not affect the main conclusions of this work.

The convergence of the energy loss with respect to the finite size effect reflects convergence of the force exerted on the HCI, as can be seen in the left panel of Supplementary Figure 7. This is particularly the case in the incoming path of the trajectory that predominantly determines the energy loss (the ion is basically neutralized after crossing the graphene layer). Additionally, it is worth to mention that energy conservation

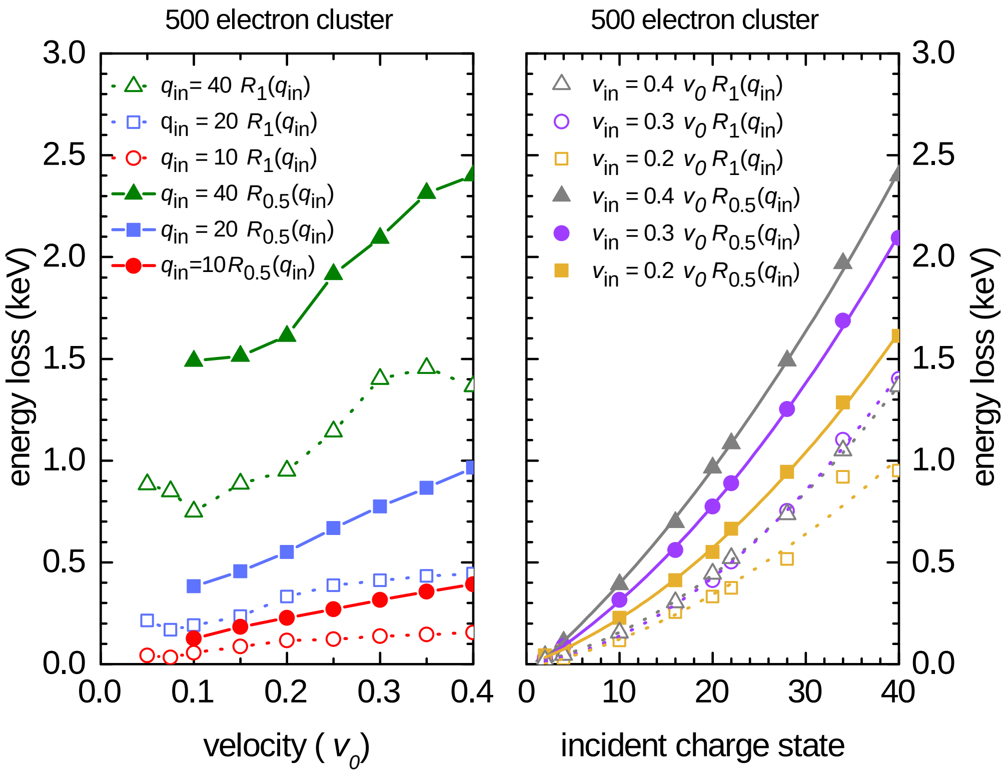

Supplementary Figure 5. Energy loss as a function of velocity and incident charge state
Energy loss as a function of HCI velocity (left panel) for incident charge states q_{in}=10, 20 and 40 and as a function of the incident charge state (right panel) for velocities v=0.88, 0.66 and 0.44 nm/fs. Both the two description of the HCI that correspond to the $R_1(q_{\mathrm{in}})$ and $R_{0.5}(q_{\mathrm{in}})$ cutoff radii for Coulomb potential (see Supplementary Equation 4) have been used. A 500 electrons jellium disk has been used to model the graphene layer. On the left panel lines are guides to the eye whereas on the right panel they are quadratic fits to the data. Solid (dash/empty) lines and symbols have been used for the dense (normal) HCI.

is perfectly fulfilled in our TDDFT calculations, as can be seen in the right panel of Supplementary Figure 7, where the two curves that correspond to the total energy and integrated value of the force along the trajectory sit one on top of the other.

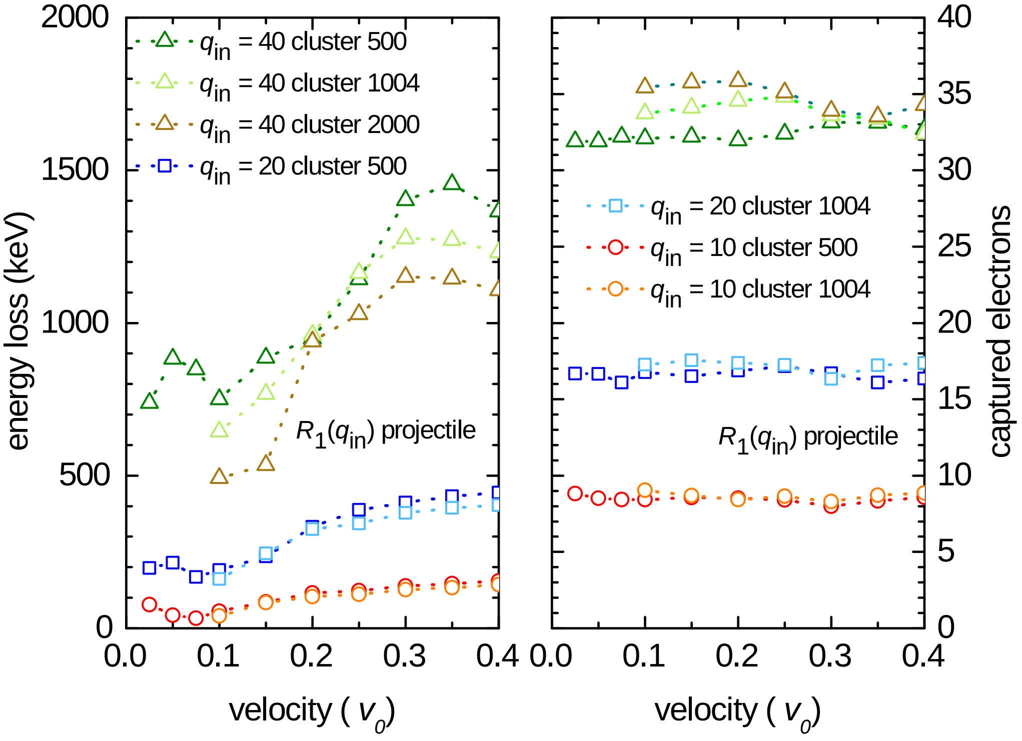

Supplementary Figure 6. Energy loss and captured electrons for different cluster sizes
Energy loss and number of captured electrons as a function of HCI velocity for incident charge states q_{in}= 10, 20 and 40 for different cluster sizes. The projectile has been modeled using $R_1(q_{\text{in}})$ cutoff radius for Coulomb potential as defined by Supplementary Equation 4. Only for the highest incident charge state $q_{\text{in}} = 40$ finite size effects are present.

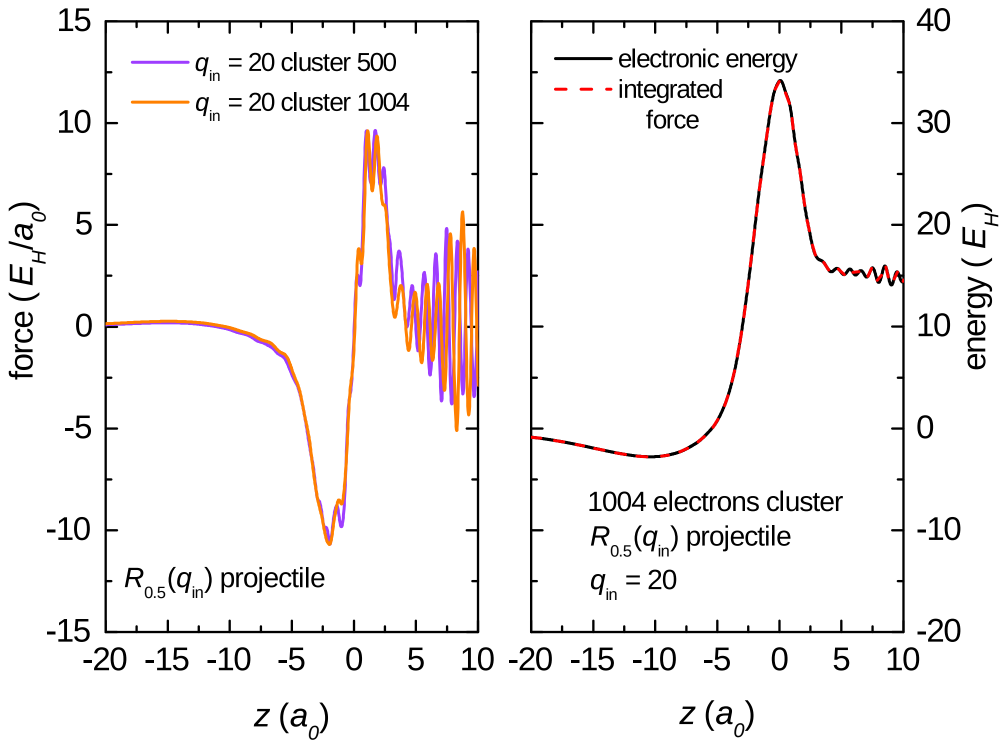

Supplementary Figure 7. Force along the trajectory of the HCI projectile

Left panel: Force exerted along the trajectory on the $q_{\mathrm{in}} = 20$ HCI projectile incident at the 500 and 1004 electrons jellium disks representing the graphene layer. The projectile has been modeled using $R_{0.5}(q_{\mathrm{in}})$ cutoff radius for Coulomb potential as defined by Supplementary Equation 4 ($R_{0.5}(20) = 0.26$ Å). The collision velocity v=0.44 nm/fs. Right panel: change of the electronic energy (solid black) and integrated force (dash red) for the same collision conditions as in left panel.

Supplementary References

1. Li, X. *et al.* Transfer of large-area graphene films for high-performance transparent conductive electrodes. *Nano Lett.* **9**, 4359–4363 (2009).
2. Kratzer, M. *et al.* Effects of polymethylmethacrylate-transfer residues on the growth of organic semiconductor molecules on chemical vapor deposited graphene. *Appl. Phys. Lett.* **106**, 103101 (2015).
3. Algara-Siller, G., Lehtinen, O., Turchanin, A. & Kaiser, U. Dry-cleaning of graphene. *Appl. Phys. Lett.* **104**, 153115 (2014).
4. Pantelic, R. S., Meyer, J. C., Kaiser, U. & Stahlberg, H. The application of graphene as a sample support in transmission electron microscopy. *Solid State Commun.* **152**, 1375–1382 (2012).
5. Zan, R., Ramasse, Q. M., Bangert, U. & Novoselov, K. S. Graphene reknits its holes. *Nano Lett.* **12**, 3936–3940 (2012).
6. Marques, M. A. & Gross, E. Time-dependent density functional theory. *Annu. Rev. Phys. Chem.* **55**, 427–455 (2004).
7. Casida, M. E. Time-dependent density-functional theory for molecules and molecular solids. *Journal of Molecular Structure: THEOCHEM* **914**, 3–18 (2009).
8. Fennel, T. *et al.* Laser-driven nonlinear cluster dynamics. *Rev. Mod. Phys.* **82**, 1793 (2010).
9. Quijada, M., Borisov, A. G., Nagy, I., Muiño, R. D. & Echenique, P. M. Time-dependent density-functional calculation of the stopping power for protons and antiprotons in metals. *Phys. Rev. A* **75**, 042902 (2007).
10. Pruneda, J. M., Sánchez-Portal, D., Arnau, A., Juaristi, J. I. & Artacho, E. Electronic stopping power in lif from first principles. *Phys. Rev. Lett.* **99**, 235501 (2007).
11. Correa, A. A., Kohanoff, J., Artacho, E., Sánchez-Portal, D. & Caro, A. Nonadiabatic forces in ion-solid interactions: the initial stages of rradiation damage. *Phys. Rev. Lett.* **108**, 213201 (2012).
12. Zeb, M. A. *et al.* Electronic stopping power in gold: the role of d electrons and the H/He anomaly. *Phys. Rev. Lett.* **108**, 225504 (2012).
13. Krasheninnikov, A. V., Miyamoto, Y. & Tománek, D. Role of electronic excitations in ion collisions with carbon nanostructures. *Phys. Rev. Lett.* **99**, 016104 (2007).
14. Bubin, S., Wang, B., Pantelides, S. & Varga, K. Simulation of high-energy ion collisions with graphene fragments. *Phys. Rev. B* **85**, 235435 (2012).
15. Ojanperä, A., Krasheninnikov, A. V. & Puska, M. Electronic stopping power from first-principles calculations with account for core electron excitations and projectile ionization. *Phys. Rev. B* **89**, 035120 (2014).
16. Zhao, S., Kang, W., Xue, J., Zhang, X. & Zhang, P. Comparison of electronic energy loss in graphene and bn sheet by means of time-dependent density functional theory. *J. Phys. Condens. Matter* **27**, 025401 (2014).
17. Mao, F., Zhang, C., Gao, C.-Z., Dai, J. & Zhang, F.-S. The effects of electron transfer on the energy loss of slow He^{2+}, C^{2+}, and C^{4+} ions penetrating a graphene fragment. *J. Phys. Condens. Matter* **26**, 085402 (2014).

18. Marinica, D., Kazansky, A., Nordlander, P., Aizpurua, J. & Borisov, A. G. Quantum plasmonics: nonlinear effects in the field enhancement of a plasmonic nanoparticle dimer. *Nano Lett.* **12**, 1333–1339 (2012).

19. Burke, K., Werschnik, J. & Gross, E. Time-dependent density functional theory: Past, present, and future. *J. Chem. Phys.* **123**, 062206 (2005).

20. Gunnarsson, O. & Lundqvist, B. Exchange and correlation in atoms, molecules, and solids by the spin-density-functional formalism. *Phys. Rev. B* **13**, 4274 (1976).

21. Lang, N. & Kohn, W. Surface-dipole barriers in simple metals. *Phys. Rev. B* **8**, 6010 (1973).

22. Brack, M. The physics of simple metal clusters: self-consistent jellium model and semiclassical approaches. *Rev. Mod. Phys.* **65**, 677 (1993).

23. Puska, M. J. & Nieminen, R. M. Photoabsorption of atoms inside C 60. *Phys. Rev. A* **47**, 1181 (1993).

24. Yannouleas, C. & Landman, U. Stabilized-jellium description of neutral and multiply charged fullerenes $C_{60}^{x\pm}$ *Chem. Phys. Lett.* **217**, 175–185 (1994).

25. Rüdel, A. *et al.* Imaging delocalized electron clouds: Photoionization of C 60 in fourier reciprocal space. *Phys. Rev. Lett.* **89**, 125503 (2002).

26. Bhardwaj, V., Corkum, P. & Rayner, D. Recollision during the high laser intensity ionization of C 60. *Phys. Rev. Lett.* **93**, 043001 (2004).

27. Madjet, M. E., Chakraborty, H. S., Rost, J. M. & Manson, S. T. Photoionization of C 60: a model study. *J. Phys. B* **41**, 105101 (2008).

28. Huismans, Y. *et al.* Macro-atom versus many-electron effects in ultrafast ionization of C 60. *Phys. Rev. A* **88**, 013201 (2013).

29. Yu, Y.-J. *et al.* Tuning the graphene work function by electric field effect. *Nano lett.* **9**, 3430–3434 (2009).

30. Yuan, H. *et al.* Engineering ultra-low work function of graphene. *Nano Lett.* **15**, 6475–6480 (2015).

31. Perdew, J. P., Tran, H. Q. & Smith, E. D. Stabilized jellium: Structureless pseudopotential model for the cohesive and surface properties of metals. *Phys. Rev. B* **42**, 11627 (1990).

32. Borisov, A. G., Kazansky, A. K. & Gauyacq, J. P. Resonant charge transfer in ion˘metal surface collisions: Effect of a projected band gap in the $H^- - Cu(111)$ system. *Phys. Rev. B* **59**, 10935–10949 (1999).

33. Borisov, A., Gauyacq, J. & Shabanov, S. Wave packet propagation study of the charge transfer interaction in the Cu (111) and-Ag (111) systems. *Surf. Sci.* **487**, 243–257 (2001).

34. Chulkov, E. *et al.* Electronic excitations in metals and at metal surfaces. *Chem. Rev.* **106**, 4160–4206 (2006).

35. Neuhasuer, D. & Baer, M. The time-dependent Schrödinger equation: Application of absorbing boundary conditions. *J. Chem. Phys.* **90**, 4351–4355 (1989).

36. Ojanperä, A., Havu, V., Lehtovaara, L. & Puska, M. Nonadiabatic ehrenfest molecular dynamics within the projector augmented-wave method. *J. Chem. Phys.* **136**, 144103 (2012).

37. Burke, K., Cruz, F. G. & Lam, K.-C. Unambiguous exchange-correlation energy density. *J. Chem. Phys.* **109**, 8161–8167 (1998).

38. Balzer, K. & Bonitz, M. Stopping dynamics of ions passing through correlated honeycomb clusters. *arXiv preprint arXiv:1602.06928* (2016).
